# The Impact of Poverty on Partner Violence Against Women Under Regional Effects: The Case of Turkey

**DOI:** 10.1177/08862605221119515

**Published:** 2022-09-01

**Authors:** Anil Eralp, Sahika Gokmen

**Affiliations:** 1 Bolu Izzet Baysal University, Bolu, Turkey; 2 Ankara Haci Bayram Veli University, Ankara, Turkey; 3 Uppsala University, Uppsala, Sweden

**Keywords:** violence against women, physical violence, sexual violence, poverty, regional effect, multinomial models, intimate partner violence

## Abstract

Violence against women has been the subject of scientific literature in many fields and poverty has been one of the most important companions in this field. It can be found lots of empirical studies about violence against women for countries as Turkey too. However, regional considerations relating to people’s socioeconomic condition have not been considered in these investigations although it has been indicated that these factors are important in terms of violence against women. Therefore, the main motivation of this study to investigate the impact of poverty on partner violence against women under the regional impacts in Turkey. The multinomial logit analysis preferred since the violence against women considered under three groups which are physical, sexual violence, and never experienced. The dataset received from the Survey on Domestic Violence Against Women in Turkey which was performed by Turkish Statistical Institute (TURKSTAT). This survey is performed in both 2008 and 2014 years. For this study, the 2008 data is chosen as it carries the information of “having a green card” which is a formal demonstration of being poor. Also, NUTS 2 (Nomenclature of territorial units for statistics) regions for Turkey are considered during the analysis. Based on the general results, the poverty status and regional effects of women, showed quite different results in terms of physical and sexual violence types. The poverty has a positive effect only on physical violence, not on sexual violence. Further, all regions have an important role on physical violence, while only less developed regions have a dominant impact on sexual violence. Also, the findings show that the intimate partners’ bad habits make women more vulnerable to violence. According to the results, it can be suggested that developing policies based on regional effects and types of violence would be more effective.

## Introduction

Violence against women is a global issue that has existed for a long time in virtually every country. According to the World Health Organization (WHO, 2021), 30% of women globally had experienced violence. Furthermore, nearly one-third of women between the ages of 15 and 49 who have been in a relationship have experienced physical and/or sexual violence from a partner. As can be seen, violence against women is a major social issue that affects the entire world, including Turkey. Based on the most recent data of Organization for Economic Cooperation and Development, 38% of women experienced physical and/or sexual violence by their intimate in 2019 and with this information, Turkey is the 26th among 129 countries (OECD, 2019).

According to WHO’s risk scheme for violence against women, the first order is taken by intimate partner’s behavioral attitudes while it is possible to see the different economic factors in second, third, and fourth orders. This scheme demonstrates that poverty has a spreading impact on violence as lots of papers in related literature referred. It is possible to examine the literature under the presence of poverty and violence against women in two part: Studies that focus on poverty headline directly (e.g., Bastos et al., 2009; Das & Roy, 2020; Kehler, 2001; Ogrodnik & Borzutzky, 2011; Slabbert, 2017; Terry, 2004; Williams, 1998) and studies that research poverty via some undirect variables besides other violence factors (e.g., Alkan et al., 2021; Ari & Aydin, 2016; Chikhungu et al., 2021; Kotan et al., 2020; Ranganathan et al., 2021). Based on all aforementioned literature, it is a common finding of the studies that violence against women is more common in poor families. Besides, it can be implied that women whose families’ have a low-income level, whose intimate partners’ education level is very low and unemployed are exposed to violence often by their intimate partners. This finding is lately approved by Kotan et al. (2020) study for Turkey. This study demonstrates that both the educational level and household income have an impact on domestic violence depending on the performed survey data by the authors.

Poverty makes living conditions more challenging and necessitates living in close quarters with high levels of violence. Furthermore, residing in these areas encourages the normalization of violence against women. Poverty is one of the most common causes of violence against women in Turkey, according to the NEE (2014) research report on domestic violence in Turkey. The reason for that, poverty can lead to males feeling powerless as they face the challenges of the big city. This situation also demonstrated that there is a regional effect on both poverty and violence. Similarly, in the NEE (2014)’s report, it is emphasized that regional difference is increasing in the context of violence against women. Based on that idea, the main motivation of this study is to investigate the effect of poverty on partner violence against women under regional impacts in Turkey. When related recent studies in the literature are examined, Ari and Aydin (2016), Kizilgol and Ipek (2018), Kotan et al. (2020), and Alkan et al. (2021) considered the violence against women in Turkey from different perspectives, but none of these studies examined the effect of regions, except Ari and Aydin (2016). Ari and Aydin (2016) examine various major regions, but this study excludes poverty as a factor and the analysis is based on 2008 data.

In the present study, the impact of poverty on partner violence against women investigate under the consideration of NUTS 2 (Nomenclature of territorial units for statistics) regions for Turkey.^1^ The reason for taking into consideration the NUTS 2 regions is that this regional classification represents the territorial division in socioeconomic ways. For the empirical analysis, the multinomial logit analysis preferred as partner violence has been taken into considered under three groups, which are physical, sexual violence, and never experienced. The dataset received from the Survey on Domestic Violence Against Women in Turkey which was performed by Turkish Statistical Institute (TURKSTAT). The 2008 data is chosen for the analysis as it carries the information about being poor.

Based on all the aforementioned information, the main hypotheses of this research are the following:

Hypothesis 1: Poverty of women has an effect on the sorts of partner violence that women face.
Hypothesis 2: The regions of Turkey have an effect on the sorts of partner violence that women face.
Hypothesis 3: The socioeconomic status of women has an effect on the sorts of partner violence that women face.
Hypothesis 4: The socioeconomic status of women’s partners has an effect on the sorts of partner violence that women face.
Hypothesis 5: The “bad habits” of women’s partners has an effect on the sorts of partner violence that women face.

Based on this, the further parts of the study are organized as follows; the second section summarizes the Multinomial Logit Model while the third section introduces the dataset and the variables used in the analysis. Also, the results of the empirical analysis are demonstrated in Section “The Results of Empirical Analysis” and finally, the conclusions and recommendations are discussed in Section “Conclusion and Discussion.”

## Multinomial Logit Model

In classical regression analysis, the dependent variable is usually defined as a continuous variable. However, for some research, the dependent variable can be categorical either. The well-known Logistic Model is used when the response variable has only two categories. If the qualitative response variable has more than two categories, the Multinomial Logit Model (MNLM), an extended version of binary logit model, can be considered to analyzing process. MNLM is actually an extension of the Logit Model and the categories may be involved in the ranking.

MNLM does not require linearity, normality, and homoscedasticity assumptions while Multinomial Probit Model (MNPM), which can also be used as an alternative to MNLM, the errors must have a normal distribution (Greene, 2020, p. 867). Therefore, MNLM is generally preferred more in empirical studies as it does not require an assumption. Further, in MNLM, there is no restriction on independent variables’ measurement level and so, quantitative and qualitative independent variables can be included in the model. The essential thing to keep in mind is that in MNLM, each cell in the cross tables to be produced between the response variable and the explanatory variables should have at least five observations.

MNLM can be considered as simultaneous estimates of binary logit models for all possible comparisons of categories and it can be expressed as below,

(1) $$\mathrm{Prob}\;\left(Y_{i}=j|X_{i}\right)=\frac{\exp\;\left({x^{\prime }}_{ij}\theta \right)}{\sum_{j=1}^{J}\exp\;\left({x^{\prime }}_{ij}\theta \right)}$$

where *j* represents the number of categories that the response variable (*Y*) has and *i* indicates the number of observations while *x* and θ indicate the explanatory variables and parameters vector respectively (Greene, 2020, p. 868). Equation (1) demonstrates the probabilities of each category under the estimation of MNLM with maximum likelihood.

Even MNLM looks flexible in terms of assumptions, it needs to be provided the assumption of independence of irrelevant alternatives (IIA). This assumption means that the odds need to be statistically independent from the categories of the response variable. Long and Freese (2001) define this assumption as the alternatives (categories) of the response variable are independent whilst Benson et al. (2016) define it as the relative probability of choosing one of the two categories is independent of any additional alternatives.

Let’s take this study as an example; the categories are defined as the types of violence against women and it is assumed that the probabilities of that the partner commits violence against women and does not are equal ( $P_{\mathrm{NV}}=P_{V}$ , respectively). The violence is described under the categories of physical and sexual. IIA assumption corresponds to meaning that the partner does not make a distinction between the types of violence if he commits violence. The relative probability of preference between non-violence and violence will change, not be $P_{\mathrm{NV}}/P_{V}=1$ . In this case, the estimated parameters will differ according to the way in which the categories of the response variables are determined. To prevent this, the IIA assumption must be met. Hausmann test developed by Hausman and McFadden (1984) to test the IIA assumption by using Chi-square distribution (Cameron & Trivedi, 2005). This test compares the restricted model estimates produced by removing at least one of the response variable categories with the unrestricted model estimates that cover all response variable categories (Long, 1997, p. 184). The IIA assumption is invalid if the difference between these two models is statistically significant. After the assumption is satisfied, MNLM can be estimated with maximum likelihood.

## Dataset and Variables

The dataset used in this study’s empirical analysis is from TURKSTAT’s Survey on Domestic Violence Against Women in Turkey. Despite the fact that this survey was conducted in both 2008 and 2014, the 2008 data is utilized for this analysis. The reason for this is that the 2008 dataset incorporates data on both poverty and regional information based on NUTS 2. In this context, the dataset can be used to reflect regional differences in violence against woman in Turkey.

According to the related dataset, some observations are excluded from the dataset due to missing observations and/or unanswered questions. In addition, the data of women who have been exposed to both physical and sexual violence in the variable of violence against women are excluded from the dataset in order to provide the IIA assumption. Hereby, 8,035 women between the ages of 15 and 59 had their information consisted.^2^

To explain the mainframe of the dataset, Table 1 is prepared to list the variables, their categories, and their frequencies. Based on the main features of the dataset, only 68.04% of the women in this sample confirmed that they had never been experienced violence and the remaining women had been exposed to at least one form of physical and sexual violence.

**Table 1.** Frequencies of Variables Explored in this Study.

	Frequency	%	Cumulative %
Types of violence			
Never experienced	5,467	68.04	68.04
Physical	2,313	28.79	96.83
Sexual	255	3.17	100
Poverty			
Have green card	1,165	14.50	14.50
Other	6,870	85.50	100
NUTS regions			
TR1-Istanbul	549	6.83	6.38
TR2-West Marmara	636	7.92	14.75
TR3-Aegean	634	7.89	22.64
TR4-East Marmara	627	7.80	30.44
TR5-West Anatolia	723	9.00	39.44
TR6-Mediterranean	680	8.46	47.90
TR7-Central Anatolia	655	8.15	56.05
TR8-West Black Sea	535	6.66	62.71
TR9-East Black Sea	617	7.68	70.39
TRA-Northeast Anatolia	694	8.64	79.03
TRB-Central East Anatolia	778	9.68	88.71
TRC-Southeast Anatolia	907	11.29	100
Age of women			
15–25	1,661	20.67	20.67
26–35	2,695	33.54	54.21
36–45	1,887	23.48	77.70
46–59	1,792	22.30	100
Education level of women			
University and above	563	7.01	7.01
High school	1,349	16.79	23.80
Primary (8 years)	762	9.48	33.28
Primary (5 years)	4,006	49.86	83.14
No education	1,355	16.86	100
Responded currently working (women)			
Working	702	8.74	8.74
Not working	7,333	91.26	100
Education level of partner			
University and above	1,158	14.41	14.41
High school	2,061	25.65	40.06
Primary (8 years)	1,132	14.09	54.15
Primary (5 years)	3,351	41.71	95.86
No education	333	414	100
Responded currently working (men)			
Working	5,605	69.76	69.76
Not working	2,430	30.24	100
Partner takes alcohol and drug			
No	6,570	81.77	81.77
Yes	1,465	18.23	100
Partner gambles			
No	7,937	98.78	98.78
Yes	98	1.22	100
Partner is having an affair			
No	7,678	95.56	95.56
Yes	357	4.44	100
Total	8,035	100	

*Note*. NUTS 2 = Nomenclature of territorial units for statistics.

One of the most important variables is poverty in this study. The question of “having a green card” is used to determine the concept of poverty which has 14.50% of the sample. Green card users, according to Turkish law, are those “who are residing in Turkey, who are not covered by any social security institution, whose monthly income is to be determined within the framework of the procedures and principles stipulated by the Law and this Regulation, or whose income share in the household is the spouse of the minimum wage determined according to Labor Law No. 4857 dated 22/5/2003; or do not work in an income-generating job.”^3^ Therefore, green card holders are given to people who are not under any social security and are unable to cover their health expenses. For this reason, in this study, the distinction of whether people are poor or not is determined according to whether they have a green card or not.

The other important factor that examined in this study is the regions. When we look at Table 1, it is seen that the distribution of the observations to the regions has similar proportions. Other variables such as education level, age, and using alcohol situations are guided by previous literature (Alkan et al., 2021; Ari & Aydin, 2016; Chikhungu et al., 2021; Kizilgol & Ipek, 2018; Ranganathan et al., 2021). Among these variables, one of the most important frequencies belongs to the “currently working” situation. The rate of women actively working in a job is 8.74% while partners’ is 69.76%. However, considering the position of the women in their work, it is seen that they are irregular and unpaid family workers. It is essential to highlight that currently working women’s frequency is relatively lower than our expectation although the rate of women with at least high school graduates is 23%. Even the ratio of having a university or higher educational level is almost twice for men compared with women, 18.23% of them uses alcohol and drug, 1.22% of them gambles, and 4.44% of them cheating on their partners.

## The Results of Empirical Analysis

The main aim of this study is to investigate the impact of poverty on partner violence against woman under the consideration of NUTS 2 regions for Turkey. For the empirical analysis, the multinomial logit analysis preferred as partner violence has been taken into consideration under three groups.

Before discussing the results of MNLM, it is necessary to check the frequency table of the response variable (See Table 1) whether the frequencies are large enough (especially if they are zero frequencies). According to Table 1, it is seen that there is no cell that has less than five observations in the types of violence. In addition, the cross-frequency tables of the response variable with explanatory variables (See Table 2) are examined. It can be seen that from Table 2, all frequencies are enough large and there is a significant relationship (*p* < .01) between all the explanatory variables and the response variable according to the results of chi-square tests. Based on this, it can be clearly said that the data set is suitable for MNLM.

**Table 2.** Cross Tabs and Pearson Chi-Square Tests for Violence and Explanatory Variables.

	No experienced (%)	Physical (%)	Sexual (%)	Chi-square
Poverty				
Have green card	4,787 (59.58)	1,874 (23.32)	209 (2.60)	58.7643 (0.000)
Other	680 (8.46)	439 (5.46)	46 (0.57)	
NUTS regions				
TR1-Istanbul	395 (6.58)	419 (1.21)	5 (0.12)	177.11525 (0.000)
TR2-West Marmara	529 (4.92)	97 (1.85)	10 (0.06)	
TR3-Aegean	473 (5.89)	146 (1.82)	15 (0.19)	
TR4-East Marmara	455 (5.66)	161 (2.00)	11 (0.14)	
TR5-West Anatolia	475 (5.91)	230 (2.86)	18 (0.22)	
TR6-Mediterranean	474 (5.90)	190 (2.36)	16 (0.20)	
TR7-Central Anatolia	413 (5.14)	217 (2.70)	25 (0.31)	
TR8-West Black Sea	350 (4.36)	163 (2.03)	22 (0.27)	
TR9-East Black Sea	436 (5.43)	149 (1.85)	32 (0.40)	
TRA-Northeast Anatolia	436 (5.43)	227 (2.83)	31 (0.39)	
TRB-Central East Anatolia	488 (6.07)	255 (3.17)	35 (0.44)	
TRC-Southeast Anatolia	543 (6.76)	329 (4.09)	35 (0.44)	
Age of women				
15–25	1,309 (16.29)	303 (3.77)	49 (0.61)	126.8254 (0.000)
26–35	1,808 (22.50)	802 (9.98)	85 (1.06)	
36–45	1,227 (15.27)	601 (7.48)	59 (0.73)	
46–59	1,123 (13.98)	607 (7.55)	62 (0.77)	
Education level of women				
University and above	489 (6.09)	65 (0.81)	9 (0.11)	250.4991 (0.000)
High school	1,047 (13.03)	267 (3.32)	35 (0.44)	
Primary (8 years)	553 (6.88)	190 (2.36)	19 (0.24)	
Primary (5 years)	2,607 (32.45)	1,266 (15.76)	133 (1.66)	
No education	771 (9.60)	525 (6.53)	59 (0.73)	
Responded currently working (women)				
Working	444 (5.53)	222 (2.76)	36 (0.45)	14.0069 (0.001)
Not working	5,023 (62.51)	2,091 (26.02)	219 (2.73)	
Education level of partner				
University and above	941 (11.71)	185 (2.30)	32 (0.40)	201.3040 (0.000)
High school	1,506 (18.74)	506 (6.30)	49 (0.61)	
Primary (8 years)	764 (9.51)	330 (4.11)	38 (0.47)	
Primary (5 years)	2,061 (25.65)	1,166 (14.51)	124 (1.54)	
No education	195 (2.43)	126 (1.57)	12 (0.15)	
Responded currently working (men)				
Working	3,868 (48.14)	1,562 (19.44)	175 (2.18)	8.1497 (0.017)
Not working	1,599 (19.90)	751 (9.35)	80 (1.00)	
Partner takes alcohol and drug				
No	4,523 (56.29)	1,833 (22.81)	214 (2.66)	33.0515 (0.000)
Yes	944 (11.75)	480 (5.97)	41 (0.51)	
Partner gambles				
No	5,423 (67.49)	2,265 (28.19)	249 (3.10)	24.5802 (0.000)
Yes	44 (0.55)	48 (0.60)	6 (0.07)	
Partner is having an affair				
No	5,317 (66.17)	2,120 (26.38)	241 (3.00)	120.7516 (0.000)
Yes	150 (1.87)	193 (2.40)	14 (0.17)	

*Note*. NUTS 2 = Nomenclature of territorial units for statistics.

The results of the MNLM and IIA assumption test are demonstrated in Table 3, which includes the probabilities of physical and sexual violence in each column. Statistically significant results (*p* < .05) of the explanatory variables on violence types according to the reference category are written in bold.^4^ Before evaluating the model prediction results, the validity of the IIA hypothesis needs to be tested. The Hausman test results demonstrates that the types of violence are statistically independent from each other (See Table 3) as it is expected to be.

**Table 3.** Results of Multinomial Logistic Regression Results on Type Violence: Relative Risk Ratios (95% Confidence Interval).

Variables	Physical	Sexual
Poverty (other)		
Have green card	1.22 (1.05, 1.43)*	0.97 (0.67, 1.41)
NUTS regions (TR2-West Marmara)		
TR1-Istanbul	2.24 (1.67, 3.02)*	0.71 (0.24, 2.12)
TR3-Aegean	1.85 (1.38, 2.48)*	1.83 (0.81, 4.13)
TR4-East Marmara	2.07 (1.55, 2.77)*	1.35 (0.56, 3.22)
TR5-West Anatolia	3.26 (2.46, 4.30)*	2.36 (1.07, 5.20)*
TR6-Mediterranean	2.34 (1.76, 3.11)*	1.91 (0.85, 4.28)
TR7-Central Anatolia	3.15 (2.37, 4.18)*	3.42 (1.61, 7.27)*
TR8-West Black Sea	2.55 (1.90, 3.42)*	3.30 (1.54, 7.10)*
TR9-East Black Sea	2.17 (1.62, 2.92)*	4.33 (2.09, 8.97)*
TRA-Northeast Anatolia	3.17 (2.38, 4.22)*	4.19 (1.98, 8.85)*
TRB-Central East Anatolia	3.41 (2.57, 4.52)*	4.35 (2.08, 9.11)*
TRC-Southeast Anatolia	3.62 (2.75, 4.78)*	3.62 (1.72, 7.61)*
Age of women (15–25)		
26–35	1.81 (1.55, 2.12)*	1.15 (0.79, 1.66)
36–45	1.88 (1.58, 2.23)*	1.09 (0.72, 1.65)
46–59	2.15 (1.79, 2.58)*	1.28 (0.83, 1.99)
Education level of women (university and above)		
High school	1.75 (1.28, 2.39)*	1.87 (0.86, 4.08)
Primary (8 years)	2.11 (1.50, 2.97)*	1.72 (0.72, 4.10)
Primary (5 years)	2.44 (1.79, 3.33)*	2.38 (1.09, 5.18)*
No education	2.66 (1.89, 3.74)*	2.73 (1.18, 6.32)*
Working status of women (not working)		
Working	1.15 (0.96, 1.37)	1.78 (1.22, 2.59)*
Education level of partner (university and above)		
High school	1.43 (1.16, 1.77)*	0.77 (0.47, 1.25)
Primary (8 years)	1.51 (1.20, 1.91)*	1.06 (0.62, 1.80)
Primary (5 years)	1.81 (1.46, 2.24)*	1.20 (0.74, 1.95)
No education	1.51 (1.09, 2.08)*	0.89 (0.41, 1.94)
Working status of partner (working)		
Not working	1.04 (0.93, 1.17)	1.06 (0.79, 1.42)
Partner takes alcohol and drug (no)		
Yes	1.64 (1.43, 1.88)*	1.21 (0.84, 1.75)
Partner gambles (no)		
Yes	1.65 (1.06, 2.57)*	2.31 (0.95, 5.65)**
Partner is having an affair (no)		
Yes	2.87 (2.28, 3.62)*	1.91 (1.08, 3.40)*
Constant	0.02 (0.01, 0.03)	0.10 (0.00, 0.02)
IIA test		
Physical violence is excluded (*p*-value)		28.74 (0.4786)
Sexual violence is excluded (*p*-value)	33.69 (0.2509)	

Note. IIA = independence of irrelevant alternatives; NUTS 2 = Nomenclature of territorial units for statistics.

* *p* < 0.05.

** *p* < 0.10.

According to the MNLM analysis results depend on the related data, poverty related results examined initially. Based on these results, it has been observed that poor women are more open to physical violence, under the assumption that the poverty situation of women remains the same. Furthermore, it is shown that disadvantaged women (elderly and with low levels of education) have a significant risk of being subjected to both types of violence. This research indicates that, the risk of exposure to domestic violence decreases in the case of not being poor (i.e., not having a green card), with the observation that domestic violence increases in families under financial pressure. Domestic violence, according to Renzetti and Larkin (2009, pp. 1–2), might result in certain financial struggles. Poverty and women in the disadvantaged group, in particular, are projected to be less likely to access social assistance and protect themselves. As a result, poverty may make individuals feel alone in the fight against domestic violence. Financially more secure women can also hide their violence from public observation, as Renzetti and Larkin (2009, pp. 2, 4) point out, by living in a hotel rather than a battered women’s shelter. Additionally, men partners may use violence against women to hide their failings as breadwinners. When the effect of the partner’s education level and work status on violence are analyzed, it is clear that the woman’s low education and unemployment that create a greater risk than the man’s low education and unemployment. Finally, this research shows that, based on the available data, poverty has a substantial impact only on physical violence, which is one of the types of violence against women. More detailed analysis can be made by applying mixed methods in further studies to investigate these topics together in the context of violence against woman.

On the other hand, the main reason for the non-significant impact of poverty on sexual violence may explain by the regulation of green card. According to the regulation, only one family member can be an employee to have a green card.^5^ This kind of poverty contains really very poor people. In this study, women who have a job is defined in two headlines: unpaid family worker and irregular worker. To sum up, even if the woman or the man has a job, women may not prefer to define/explain violence as sexual since they do not have neither any alternative life choices nor enough awareness. Moreover, in the literature, similarly, Dalal’s (2011) study demonstrates that family that have only female-worker had more intimate personal violence exposure than only male-worker families. Besides, some researches in the presence of sexual violence, for example, Aydin et al. (2009) indicated that sexual violence can be originated from mostly colleagues and superiors rather than their partners.

There are significant points to interpret based on the region’s implications. In general, the regional coefficients of sexual violence are quite large compared to physical violence’s. This shows that there is a significant difference both in terms of the type of violence and regionally. If we looked in detail, there are statistically significant positive sided effects on physical violence for all regions with the largest effect on Southeast Anatolia and the lowest effect on the Aegean. This situation is also consistent with the related literature. The main reason for this can be the difference in development levels between Turkey’s west and east areas. A similar pattern can be seen in Table 2. Further, the regional effects of sexual violence differ from physical violence. In general, mostly less developed regions^6^ have statistically significant effects on sexual violence. To get further interpretation about regional effects on sexual violence, the question of “Responden Agrees: Obliged to Sexual Intercourse” is taken attention in the related survey study which the frequencies are given in Table 4. Regional approaches on questionnaires that have “yes” answers have a similar pattern with the regional information in Table 3. This further information raises the idea that there is a question mark about the perception of sexual violence.

**Table 4.** The Frequencies of the Answers on “Responden Agrees: Obliged to Sexual Intercourse.”

Regions	Agree	Disagree	No idea	Missing
TR1-Istanbul	22.22	76.32	1.46	0.00
TR3-Aegean	26.57	72.01	1.26	0.16
TR4-East Marmara	25.24	73.97	0.79	0.00
TR5-West Anatolia	25.84	72.57	1.44	0.16
TR6-Mediterranean	26.97	72.48	0.55	0.00
TR7-Central Anatolia	28.24	70.00	1.62	0.15
TR8-West Black Sea	30.84	66.72	2.29	0.15
TR9-East Black Sea	28.79	70.28	0.75	0.19
TRA-Northeast Anatolia	27.88	70.83	1.3	0.00
TRB-Central East Anatolia	38.76	60.37	0.86	0.00
TRC-Southeast Anatolia	36.12	61.44	1.93	0.51
TR1-Istanbul	33.63	64.83	1.43	0.11

When we examine the other results, although the age of the woman has a statistically significant effect on physical violence in all categories, it does not seem to have a statistically significant effect on sexual violence. In terms of physical violence, when the age of the woman increases, it is seen that the probability of being exposed to physical violence increases. Furthermore, as expected from the relevant research (e.g., Basar & Demirci, 2018; Sen & Bolsoy, 2017), the probability of physical violence increases as the woman’s education level declines, despite the fact that physical violence remains an issue for women of all educational levels. However, sexual violence has a statistically significant effect on women who have not received any education or only primary school (5 years) education, and having no education has the biggest impact on sexual violence. In the end, it can be clearly highlighted that having lower education levels have higher physical/sexual violence risk than the others.

If we evaluate the situation from the partners’ side, the education level of the woman’s partner has an increasing effect on physical violence while it has no effect on sexual violence. In the context of physical violence, with those who have graduated from primary school (5 years) having the greatest impact on the risk of violence. On the contrary, the unemployed partner does not have a statistically significant effect on both physical and sexual violence. The use of alcohol and drugs by the partner has an increasing effect on physical violence, while it has not on sexual violence. However, the partner’s gambling has a positive and significant effect on both physical and sexual violence, and the impact of sexual violence is greater than physical violence. Similarly, the partner’s having an affair with someone else has a statistically significant and increasing effect on both physical and sexual violence. Physical violence has a more significant impact rather than others.

## Conclusion and Discussion

This study investigates the effect of poverty on intimate partner violence against women through the NUTS 2 regions for Turkey as the violence against women is unacceptable according to the Ministry of Family and Social Policies in Turkey. However, a decreasing trend has not been observed yet about violence against women. Through the findings of multinomial logit analysis, this study intends to provide policymakers with new information based on poverty and region.

Based on the general results, the poverty status and regional effects of women, which are the focus of the study, showed quite different results in terms of physical and sexual violence types. The poverty status of women has an important positive effect only on physical violence. While all regions have an increasing effect on physical violence, only for some regions that are considered as underdeveloped, have a positive sided effect on sexual violence. This can be considered as a sign of determining the focal points for the prevention of forms of violence. Especially when the question of “Responden Agrees: Obliged to Sexual Intercourse” is considered, the results bring the question mark about the consciousness of the definition of sexual violence for women. If the education level, age, and employment status of women are evaluated together, it can be interpreted that women who are in the most disadvantaged situation (lower education level, lower age, and non-employee) are more open to violence. The results demonstrate the conclusion like the having a job increases the risk of sexual violence since the definition of jobs are not regular/official jobs in here. As a result, expanding information activities on human and women’s rights can be taken as a policy recommendation in these regions while preventing poverty can be taken as a solution for physical violence. Moreover, it is seen that it is necessary to carry out awareness-raising activities against violence throughout Turkey.

On the partner’s side, the level of education and alcohol/drug behavior of a woman’s partner have a significant impact on physical violence but have no impact on sexual violence. The decrease in the education level of the partner increases the risk of physical violence. However, gambling and having an affair increase the risk of both violence types. In short, it can be interpreted that the low education level of the partner, alcohol/drug use, and bad habits influence violence.

The conclusions of this study are based on the 2008 data of the Survey on Domestic Violence Against Women in Turkey. The findings of the 2014 study, which is considered a continuation of this one, and the outcomes of the two studies are generally similar (NEE, 2014). In this context, it has been observed that poor women are more open to physical violence, under the assumption that the poverty situation of women continues in a similar way. However, it has been determined that disadvantaged women (with low education level, low age, and non-working), have a high risk of being exposed to both types of violence. Besides, working women (irregular and unpaid family worker) are at a higher risk of experiencing sexual violence. As a result, for women, support policies like employment courses and microloan support can be used to eliminate poverty and/or women may be granted positive privileges if they have been exposed to violence in current programs. In addition, incentive programs can be implemented to improve the educational status of women exposed to violence.

To summarize, it is thought that developing policies to raise awareness of human rights and women’s rights, especially among poor and low-educated women in underdeveloped regions, will help prevent sexual violence. Moreover, as the effect of poverty on physical violence is determined as independent of the region, it is recommended to make macro regulations in order to raise awareness of the society in general on human and women’s rights, and to prevent activities that involve violence.

## Appendix

**Table A1.** Regional Distribution of Green Card Ownership.

Regions	Not Green Card Ownership (%)	Green Card Ownership (Poverty) (%)	Total (%)
TR1-Istanbul	611 (7.60)	25 (0.31)	636 (7.92)
TR3-Aegean	531 (6.61)	18 (0.22)	549 (6.83)
TR4-East Marmara	608 (7.57)	26 (0.32)	634 (7.89)
TR5-West Anatolia	596 (7.42)	31 (0.39)	627 (7.80)
TR6-Mediterranean	659 (8.20)	64 (0.80)	723 (9.00)
TR7-Central Anatolia	611 (7.60)	69 (0.86)	680 (8.46)
TR8-West Black Sea	579 (7.21)	76 (0.95)	655 (8.15)
TR9-East Black Sea	488 (6.07)	47 (0.58)	535 (6.66)
TRA-Northeast Anatolia	551 (6.86)	66 (0.82)	617 (7.68)
TRB-Central East Anatolia	451 (5.61)	243 (3.02)	694 (8.64)
TRC-Southeast Anatolia	545 (6.78)	233 (2.90)	778 (9.68)
TR1-Istanbul	640 (7.97)	267 (3.32)	907 (11.29)
Total	6,870 (85.50)	1,165 (14.50)	8,035 (100.00)

*Note*. Pearson χ^2^(11) = 817.9150 (*p*-value = 0.0000).

## Notes

1. Since 2002 Turkey has been using NUTS classification in order to produce statistics at the regional level and to develop regional policies for economic and social problems at the regional level. For Turkey’s regions, governmental institutions conduct socioeconomic development studies (SEGE) to determine the socioeconomic situation. Many indicators from demographics, employment, education, health, industry, agriculture, finance, and infrastructure are employed in that sense to reflect regional development differences.

2. The following observations are excluded from the analysis: 1,073 from the violence against women variable, 5 from the poverty variable, 2 from the women’s education level variable, 1,827 from the women’s employment status variable, 56 from the partner’s education level variable, 92 from the partner’s employment status variable, 47 from the partner’s drug use variable, 37 from the partner gambling variable, and 514 from the partner cheating variable. In order to provide the IIA assumption, 1,538 observations based on both physical and sexual violence are removed from the analysis.

3. T.R. Presidential Legislation Information System, https://www.mevzuat.gov.tr/mevzuat?MevzuatNo=4846&MevzuatTur=7&MevzuatTertip=5, achieved: December 2021.

4. The explanatory variable “partner gambles” is statistically significant at the level of 0.10% on sexual violence.

5. chrome-extension://efaidnbmnnnibpcajpcglclefindmkaj/https://www.mevzuat.gov.tr/File/GeneratePdf?mevzuatNo=4846&mevzuatTur=KurumVeKurulusYonetmeligi&mevzuatTertip=5, achieved: May 2022.

6. West Anatolia, Central Anatolia, West Black Sea, East Black Sea, North East Anatolia, Central East Anatolia, South East Anatolia. While SEGE-2003 (SEGE, 2003) split Turkey into five separate regional socioeconomic development levels, SEGE-2013 (SEGE, 2013) and SEGE-2017 (SEGE, 2019) divided it into six different regional socioeconomic development levels. The findings of all three studies reveal that Turkey is developing faster in the west than it is in the east. To support this idea for the current dataset, Table A1 in Appendix section demonstrates the differences between the regions and poverty.
